# A Rare Case of Antepartum Spontaneous Septostomy in a Monochorionic Diamniotic Twin Pregnancy

**DOI:** 10.1155/2012/748614

**Published:** 2012-09-16

**Authors:** Rati Chadha, Ian R. Lange, Lisa Bratz, Stephanie L. Cooper, Anne Roggensack, Jo-Ann Johnson

**Affiliations:** ^1^Division of Maternal-Fetal Medicine, Department of Obstetrics and Gynecology, Foothills Medical Center, Calgary, AB, Canada T2N 2T9; ^2^Department of Diagnostic Imaging, Foothills Medical Center, Calgary, AB, Canada T2N 2T9

## Abstract

Spontaneous septostomy in a monochorionic diamniotic twin pregnancy is a rare phenomenon. We present a case of monochorionic diamniotic twin pregnancy with an intact dividing membrane seen in the 1st half of the pregnancy. At 26 weeks, when she was assessed for preterm contractions, the dividing membrane was not documented, which suggested spontaneous septostomy. There had been no invasive procedures during the pregnancy. She subsequently delivered at 29 weeks, secondary to preterm labor. No dividing membrane was noticed at the time of caesarian section. Spontaneous septostomy can complicate the management of monochorionic diamniotic twins by creating a pseudomonoamniotic environment resulting in cord entanglement, and difficulty in the diagnosis and management of twin-twin transfusion syndrome. We believe that such a case should be managed as monochorionic monoamniotic twin gestation.

## 1. Background

The antenatal management of a twin pregnancy is directed, in part, by the ultrasound appearance of the dividing membrane. Monochorionic diamniotic (MCDA) twin pregnancies have a higher incidence of antenatal complications largely attributed to abnormal placentation and the potential development of the twin to twin transfusion syndrome (TTTS). Conversely, monochorionic monoamniotic (MCMA) twins, which represent approximately 1% of all twins, have the highest perinatal mortality (30–70%) [1], due to prematurity, growth restriction, congenital anomalies, vascular anastomosis, and cord entanglement [2]. Umbilical cord entanglement is known to occur in as many as 70% of MCMA twins [3].

Spontaneous septostomy of the dividing membrane (SSDM) in MCDA gestation results in a pseudomonamniotic environment, which may also result in umbilical cord entanglement. SSDM is a rare event and presents a diagnostic and management challenge. We wish to describe such a case with the findings at diagnosis and review of our management. 

## 2. A Case Report

**Figure 1:**
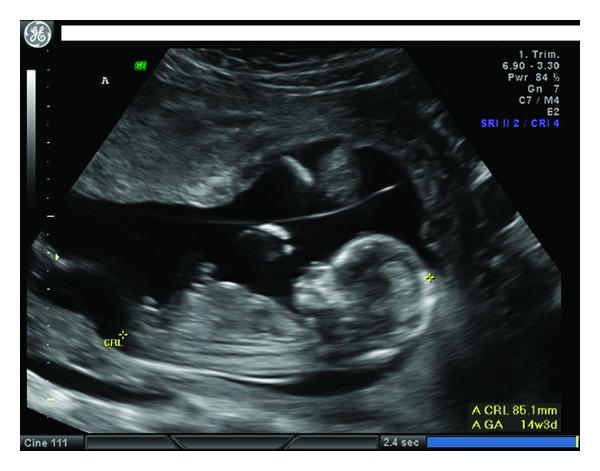
Intact membrane seen at the time of the nuchal scan.

The patient was referred to our tertiary care hospital for threatened preterm labor at a confirmed gestational age of 26 weeks, 1 day. Her first obstetrical ultrasound was a nuchal translucency assessment with aneuploidy “screen negative” results for both twins. At this examination a “T” sign, of the dividing membrane inserting into the fused posterior placenta suggested a MCDA twin pregnancy. Thereafter three further obstetrical ultrasounds confirmed the chorionicity (Figure 1). The patient was otherwise healthy, and there were no other medical or obstetrical concerns. No invasive intrauterine procedure was done during pregnancy.

**Figure 2:**
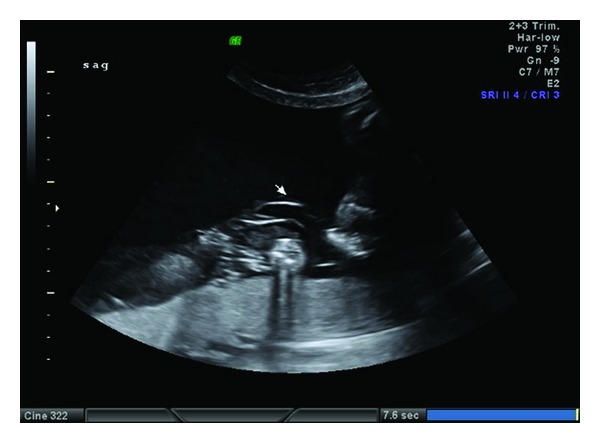
Spontaneous septostomy noted at 26 wks. Layers of membrane noted adjacent to placenta.

The patient was admitted to the hospital and was administered antenatal steroids as per the routine protocol (12 mg IM 2 doses 24 hours apart) in view of the possibility of an early delivery. Fetal heart rate monitoring on both twins reported reactive patterns. An obstetrical ultrasound confirmed a twin pregnancy with a single posterior placenta. Biophysical profile scores and fetal Doppler studies were reassuring, and there was no evidence of developing TTTS. Less than 20% growth discordance was noted on biometry. However, the twin chorionicity could not be confirmed as no dividing membrane was seen; rather folded sheets of amnion lay over the placenta (Figure 2). It was also documented that both placental cord insertions were within 3 cm of each other and that no membrane was seen separating the cord insertions. Finally, the appearance and adjacency of both twin fetal movements established a diagnosis of SSDM. It was also documented that the patient was aware of increased fetal movement in the intervals since admission. 

The patient was informed of these findings and was managed thereafter as a MCMA twin pregnancy. Ultrasound fetal assessments were ordered on alternative days using both BPP and fetal Doppler studies, while fetal biometry was performed weekly. Fetal heart monitoring was documented on alternate days to the ultrasound assessment. All aspects of fetal monitoring remained satisfactory and there was no evidence of umbilical cord entanglement. During this admission the fetal growth profiles were charted on the 10th (twin A) and the 30th percentiles (twin B), respectively. Normal interval growth was also recorded. At the final fetal assessment, one day prior to delivery, the estimated fetal weights were Twin A, 1080 gms and twin B, 1460 gms. As the fetal positions and presentations changed during this admission the twins were identified not only by their biometry but also by the appearance of each twin's umbilical cord coiling. Twin A's cord was uncoiled while twin B's was coiled.The R. I. of Twin A's umbilical artery was slightly more than of Twin B, but still had positive end-diastolic flow. At all assessments there was normal Doppler “a” wave in the ductus venosus. 

After more than three weeks in hospital and at a gestational age of 29 weeks and 5 days, the patient became aware of increased pelvic pressure with increased uterine activity. On speculum examination, the membranes were found to be “hour glassing.” A caesarian section was therefore under taken. There was no inter-twin membrane notedat the time of delivery.The twin boys weighed 1130 grams and 1350 grams, respectively, and both Apgar scores were satisfactory. There is no documentation of cord entanglement at delivery. The placental pathology confirmed MCDA twins with acute chorioamnioitis. The membranes were found to be totally stripped off from the point of insertion in the placenta except for a small area. The cord insertions were 1 cm apart. There was evidence of vascular anastomosis.

There was an uneventful postoperative recovery and after 64 and 58 days in the special care nursery, both infants were discharged home. 

## 3. Discussion

SSDM is a rare event. In a case study by Chmait et al. [4], they found a rate of SSDM at 1.8% among a highly selected cohort of MCDA twins referred to the tertiary care center for potential fetal therapy during a 2^1/2^ year period. They described the following sonographic findings that were suggestive of SSDM, if a prior invasive procedure had not been performed: absence or disrupted dividing membrane; both fetuses occupying the same side of the dividing membrane; umbilical cord entanglement and excess amniotic fluid on both sides of the dividing membrane with features suggestive of TTTS. In our case, the membrane which was clearly visible at the three earlier ultrasounds, on admission to hospital was no longer seen. Instead, sheets of folded amnion were seen lying over the posterior placenta.

The most important concern of antepartum disruption of the intertwin dividing membrane is cord entanglement. In a review of literature [5], this was reported is 60% of cases, which is close to the risk in true MCMA twins (70%) [3]. In the “hanging noose sign,” first described byLópez Ramón Y Cajal and Ocampo Marínez [6], is the characteristic sonographic appearance of a true umbilical knot, in transverse section, surrounded by a loop of umbilical cord. This may, in MCDA twins, reflect SSDM, with umbilical cord entanglement of the fetuses in the resulting from pseudo amniotic twin gestation. However, in our case repeated ultrasound examinations and the operative findings did not reveal cord entanglement.

Invasive intrauterine procedures are the most common cause of iatrogenic antepartum rupture of the dividing membrane. In our case, similar to other reports, the etiology of spontaneous antepartum rupture of the dividing membrane is unclear. The various possible etiologies include chorioamnionitis, fetal movement, polyhydramnios, and developmental disturbances [7]. Amniotic plica suggests previous rupture with subsequent fetal irritation and degenerative processes. This appears as a ridge of diamniotic membrane across the surface of placenta diagnosed on histopathology [8]. In MCDA twins, it may also obscure the diagnosis of TTTS because of equalization of amniotic fluid and may complicate the surgical management of this condition [4]. This results from the free floating dividing membrane hampering the view of placental vascular anatomy during laser therapy. This clearly demonstrates that visualization of the intertwin-dividing membrane does not ensure against possible complications of SSDM [9]. Few cases of dizygotic twin pregnancies have also been reported with SSDM [9].

Apart from the complications secondary to a pseudoamniotic environment, SSDM can also result in preterm labor and amniotic band syndrome [10]. Theetiology of the preterm labor is difficult to tease out from the inherent risk of preterm labor as a result of multiple gestation. This was also noted in our case.

This case does highlight the need of a thorough examination of the dividing membrane, at each antenatal visit by visualizing multiple sections of the membrane, because the managements of MCDA and MCMA twins are very different.

In summary, our case clearly shows that with antepartum rupture of the intertwine membrane, the MCDA pregnancy should be followed as a MCMA pregnancy with regards to fetal surveillance, time and mode of delivery given the high perinatal mortality of MCMA pregnancies.
